# Synthesis of porous zinc-based/zinc oxide composites via sol–gel and ambient pressure drying routes

**DOI:** 10.1007/s10853-018-2138-2

**Published:** 2018-02-26

**Authors:** Xiao Han, Jonathan Harris, Lidija Šiller

**Affiliations:** School of Chemical Engineering and Advanced Materials, Newcastle University, Newcastle upon Tyne, NE1 7RU UK

## Abstract

Porous Zn-based and ZnO composites are successfully fabricated via the sol–gel process and ambient pressure drying method using hexane as the drying solvent for the reduction in capillary force during drying process. Various highly porous Zn-based phases (Zn_1_-based and Zn_5_-based) that are studied by X-ray diffraction analysis, scanning electron microscopy and transmission electron microscopy show that they contribute through heat treatment (at 200 °C) to the development of ambient pressure dried nanoporous wurtzite (hexagonal) ZnO. A macroporous flower-like structure consisting of nanosheets is observed in porous Zn-based composites, and nanoporous structure is observed within platelets of ZnO nanoparticles. Possible routes for preparing highly porous Zn-based/ZnO composites are discovered by detailing the process for ambient pressure drying synthesis of porous wurzite ZnO.

**Electronic supplementary material:**

The online version of this article (10.1007/s10853-018-2138-2) contains supplementary material, which is available to authorized users.

## Introduction

Zinc oxide, as a wide band gap semiconductor (with gap energy, *E*_g_ = 3.37 eV), is known for its dielectric and photocatalytic properties [1, 2]. Zinc oxide can form a variety of stable crystal structures, such as wurtzite and zincblende structures. The tetrahedrally coordinated oxygen in ZnO within the wurtzite (hexagonal) structure gives this material the piezoelectric and pyroelectric properties [3–5]. The pyroelectricity of hexanogal ZnO has been reported in energy application as ‘nanogenerators’ [4]. Until today, the porous ZnO has been synthesised by the supercritical drying (SCD) method [6–10]. Chen et al. [8] reported that the samples fabricated by SCD method at low temperature (40 °C) are not ZnO unless they are subsequently calcined at 400–500 °C, and their intermediate products are Zn-based (zinc hydroxide nitrate) aerogels. Until now, to the best of our knowledge there is no zinc oxide aerogels prepared via ambient pressure drying (APD) route. The sol–gel process for preparing metal oxide, including ZnO, bases on the hydrolysis with epoxide [11–13]. Synthesis of ZnO nanoparticles with propylene oxide (PPO) has been reported previously [14, 15]. The hydrolysis of zinc salt precursor relies on the acid scavenger (epoxide) through protonation of the epoxide oxygen and subsequent ring opening by the nucleophilic anionic conjugate base [14, 15]. The reaction (1) below illustrates the hydrolysis of aqua zinc nitrate salt with PPO during gelation in order to obtain the ZnO crystal [15].1


Supercritical drying (SCD) is a common drying method used to fabricate aerogels. It relies on the extraction of supercritical fluids and requires autoclave to ensure that the solvent in the pores of wet gels reaches the supercritical point. In order to use the SCD method for production of zinc oxide aerogels, including one-step SCD process, a specially designed supercritical drier is required but this introduces additional costs for the manufacturing equipment [16]. The ambient pressure drying (APD) method is an alternative in synthesis of aerogel materials [17–21]. Typically, in APD synthesis of aerogels, an organic solvent with low surface tension is utilised as the drying solvent in order to reduce the capillarity during the drying process [21, 22]. Therefore, the APD method is a more economical process compared to the SCD method and is able to operate continuously as opposed to producing batches [17]. Hexane is a well-known low-surface-tension solvent and it has been often used in the APD synthesis of silica aerogels [18–20, 23]. In this work, we also choose hexane as drying solvent because solvent combination of methanol and hexane has been previously reported in solvent exchange for aerogel preparation [23], and study different phases of porous Zn-based/ZnO composites by APD method of synthesis. The determination of different phases of Zn-based/ZnO composites is necessary in order to develop the APD method of pure ZnO aerogel materials.

## Experimental

### Preparation of materials

Propylene oxide (≥ 99.5%, PPO), methanol (≥ 99.9%), ethanol (≥ 99.8%), Zn(NO_3_)_2_·6H_2_O (98%) and hexane (≥ 98.5%) were all purchased from Sigma-Aldrich (UK) and used without any further purification. The initial sol–gel process was carried out via the synthesis method reported for supercritical dried ZnO aerogels [24]. In brief, Zn(NO_3_)_2_·6H_2_O (0.238 g) was mixed with methanol (1.25 ml) and PPO (0.465 g) for gelation in the casting mould (9/11/25 mm of diameter) for 12 h. The formed gels were washed and aged with methanol/ethanol for 5 days. Then, in the following 4 days, the solvent was replaced by hexane 4 times (24 h per time). At the end of the process, the gels were dried at 60–65 °C for 5 h in the water bath and subsequently dried to 100 °C for another 3 h in the oven with air. All the drying processes were carried out under ambient pressure. The obtained Zn-based composites in this work were heated to 200 °C for 2 days to form pure ZnO product. Zn-based (ZBAG) and ZnO (ZOAG) composites were named according to the different conditions of the preparation, as Table 1 indicates. Samples ZBAG1, ZBAG3 and ZBAG4 were prepared under the same conditions except the diameter of their casting mould was 11, 9 and 25 mm, respectively. Samples ZBAG1 and ZBAG2 were prepared under the same conditions except their initial drying temperature was 60 and 65 °C, respectively. Samples ZBAG3 and ZBAG5 were prepared under the same conditions expect the ageing solvents were methanol and ethanol, respectively. Samples ZOAG 1–3 were calcined from samples ZBAG 1–3 at 200 °C.

**Table 1:** Experimental conditions used for preparing ambient pressure dried porous Zn-based (ZBAG) and ZnO (ZOAG) composites

Sample	Diameter of mould (mm)	Ageing solvent	Initial drying temperature (°C)	Heat treatment (°C)
ZBAG1/ZOAG1	11	Methanol	60	200
ZBAG2/ZOAG2	11	Methanol	65	200
ZBAG3/ZOAG3	9	Methanol	60	200
ZBAG4	25	Methanol	60	–
ZBAG5	9	Ethanol	60	–

### Materials characterisation

All characterisations have been done at Newcastle University, UK. The PANalytical X’Pert Pro Multipurpose Diffractometer (MPD) is used for X-ray diffraction (XRD) analysis with Cu Kα X-rays. All samples were mounted on a silicon low background substrate and scans were done over the 2*θ* range 5°–120°. FEI XL30 ESEM-FEG (Environmental Scanning Electron Microscope–Field Emission Gun) was used to image the samples in high vacuum mode and 10 keV accelerating voltage. Before SEM imaging, the samples were coated with gold. Philips CM-100 TEM (transmission electron microscope) was used to image samples ZBAG1–3 and ZOAG1–3. The samples for TEM were prepared by ultrasonication of porous Zn-based/ZnO composites in de-ionised water for a prolonged time so that there were no large pieces of samples seen by the eye. The surface area of the samples ZBAG 1 and ZOAG 1 was determined from N_2_ adsorption isotherms using a Surfer system (Thermo Scientific). The surface area was calculated by measuring the amount of adsorbed nitrogen gas in a relative vapour pressure of 0.05–0.3 at 77 K by Brunauer–Emmett–Teller (BET) analysis. The conditions of synthesis have been repeated and they lead to the same results (samples ZBAG R1–R3 and R’1-R’3 were repeated under the exact same conditions as samples ZBAG 1–3, and results are shown in supporting information). Fourier transform infrared spectroscopy (FTIR) for ZOAG 1 and ZBAG 1 was analysed by Perkin Elmer Spectrum 2 with a resolution of 4 and 16 scans per sample.

## Results and discussion

**Figure 1:**
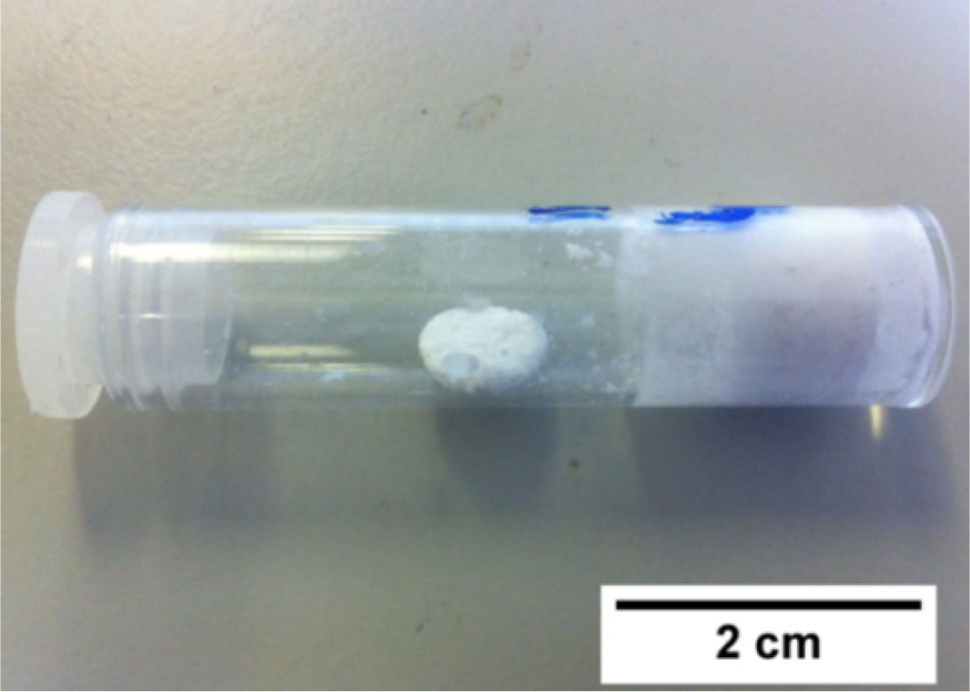
Ambient pressure dried porous Zn-based composites (ZBAG 1)

**Figure 2:**
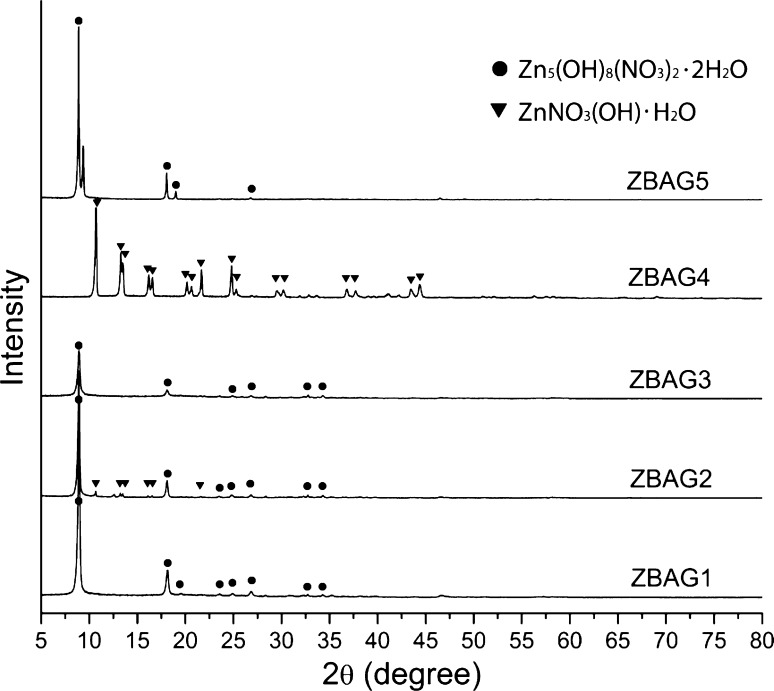
X-ray diffraction of ambient pressure dried porous Zn-based composites ZBAG1–ZBAG5

Porous Zn-based composites are obtained after drying (sample ZBAG 1 is shown in Fig. 1). Figure 2 shows the XRD analysis of ambient pressure dried porous Zn-based samples. The diffraction patterns of ZBAG1 are a single phase of Zn_5_(OH)_8_(NO_3_)_2_·2H_2_O (*PDF 01*-*072*-*0627*) (also called as Zn5) [25–28], and the diffraction patterns of ZBAG2 are a Zn_5_(OH)_8_(NO_3_)_2_·2H_2_O phase which coexists with ZnNO_3_(OH)·H_2_O (*PDF 27*-*1491*) phase (also called as Zn1) [25–28]. Zn_1_ and Zn_5_ are type I and type IIb crystal structures, respectively, which have been catalogued by Louer et al. based on different lamellar crystal structure [26]. The samples ZBAG1 and ZBAG2 are prepared by the same synthetic method except for the initial drying temperature, and they were dried for 5 h at 60 and 65 °C, respectively. The ZBAG2 samples contain the mixture of porous Zn_5_-based and Zn_1_-based composites while the ZBAG1 sample only consists of porous Zn_5_-based composites. Previous studies suggested that the Zn_1_-based and Zn_5_-based materials are generated via hydrolysis at 65 and 60 °C, respectively [25, 27]. Our conventional APD method also fabricates the same distinctive forms of zinc hydroxide nitrate at 65 and 60 °C.

The samples ZBAG1, ZBAG3 and ZBAG4 are prepared by the same synthesis method but with variation of the cross-sectional diameters of casting mould as 11, 9 and 25 mm, respectively. XRD patterns of ZBAG1 and ZBAG3 both show a single phase of Zn_5_(OH)_8_(NO_3_)_2_·2H_2_O (Zn5-based), but interestingly the XRD patterns of ZBAG4 show a single phase of ZnNO_3_(OH)·H_2_O (Zn1-based) (Fig. 2). Furthermore, the XRD patterns of ZBAG5 dried with ethanol and 9-mm casting mould reveal a single phase of Zn5 which is as the same as ZBAG3 (Fig. 2). Therefore, at a given drying temperature, the casting mould affects the formation of Zn-based products because the different evaporation surface area causes a different pressure of the solvent (hexane) inside the pores of the gels (in the enclosed vials) during the drying process.

**Figure 3:**
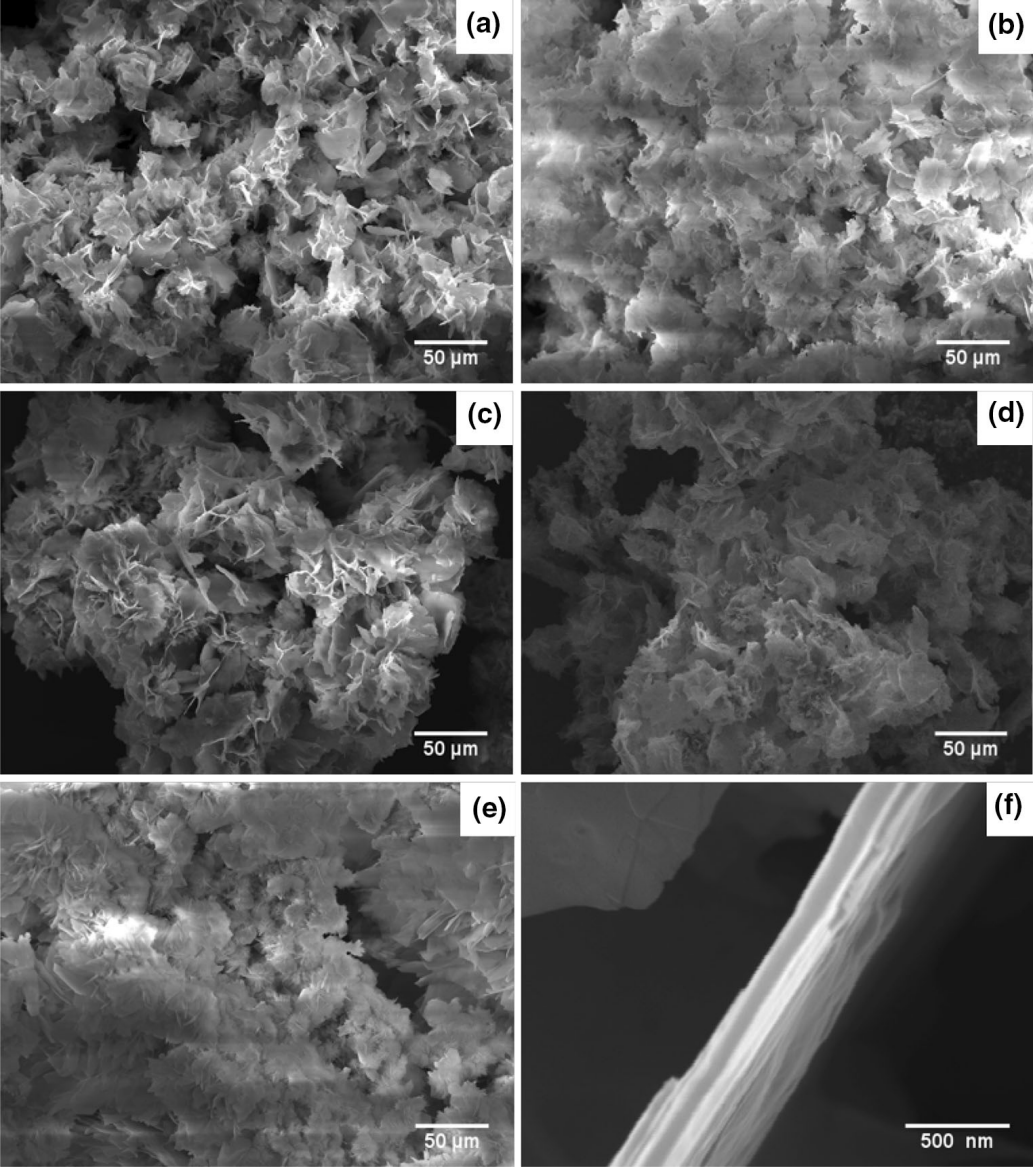
Scanning electron microscope images of ambient pressure dried porous Zn-based composites: **a** ZBAG1, **b** ZBAG2, **c** ZBAG3, **d** ZBAG4, **e** ZBAG5, **f** a captured image of the edge of the vertical nanosheets

All the SEM images of Zn-based samples ZBAG1–5 show a typical flower-like microstructure (Fig. 3a–e) which is previously reported for zinc oxide nanoparticles and supercritical dried Zn-Co aerogels [29, 30]. Moreover, it has been suggested that this structure gives good stability against the aggregation of the ZnO nanoparticles [31]. Similarly, we suggest that the macroporous (> 50 nm) flower-like structure of our porous Zn-based composites can help prevent capillary shrinkage during the ambient pressure drying process. Figure 3f shows that each plate of porous Zn-based composites is constructed of multi-layered nanosheets. Similarly multi-layered nanosheets are reported in the flower-like ZnO nanoparticles and they are grown upward from the centre [32].

**Figure 4:**
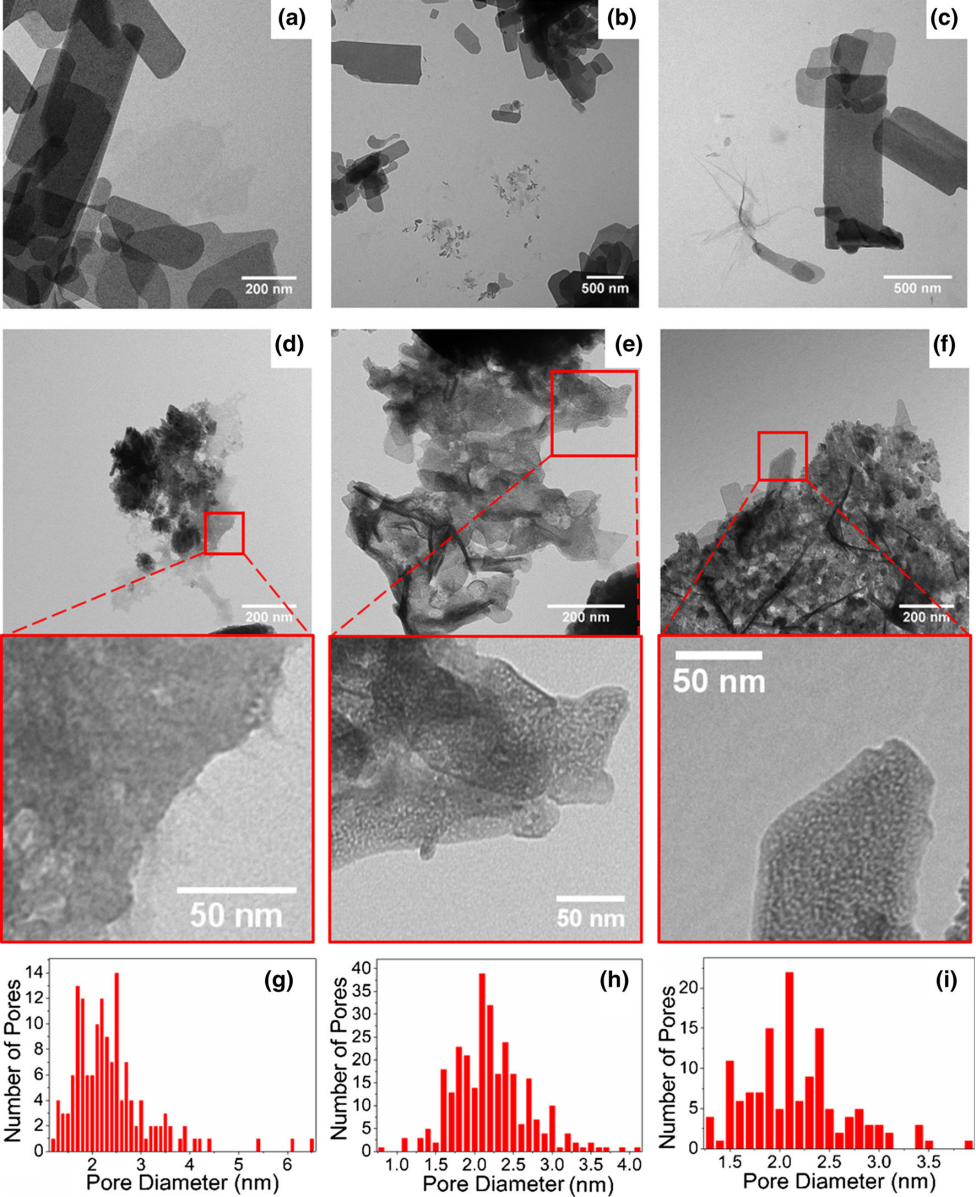
TEM images of zinc-based samples **a** ZBAG 1, **b** ZBAG 2 and **c** ZBAG 3 and zinc oxide samples **d** ZOAG 1, **e** ZOAG 2 and **f** ZOAG 3 and **g**–**i** the pore sizes distributions of zinc oxide samples 1 (analysed by 149 pores), sample 2 (analysed by 290 pores) and sample 3 (analysed by 138 pores), respectively

Figure 4a–c shows the TEM images of Zn-based samples ZBAG1–3, respectively. The nanoporous structure can be observed in Fig. 4a. Interestingly, in Fig. 4c, a radial structure is observed. The formation mechanism of the radial structure has previously been discussed in a study of flower-like ZnO nanosheets fabricated with zinc hydroxide carbonate (ZHC) precursor [33]. It has been suggested that the multi-layered nanosheets are caused by the formation of two different surfaces after nucleation of ZHC and proposed that the flower-like structure is formed due to the subsequent self-assembly of those nanosheets [33].

**Figure 5:**
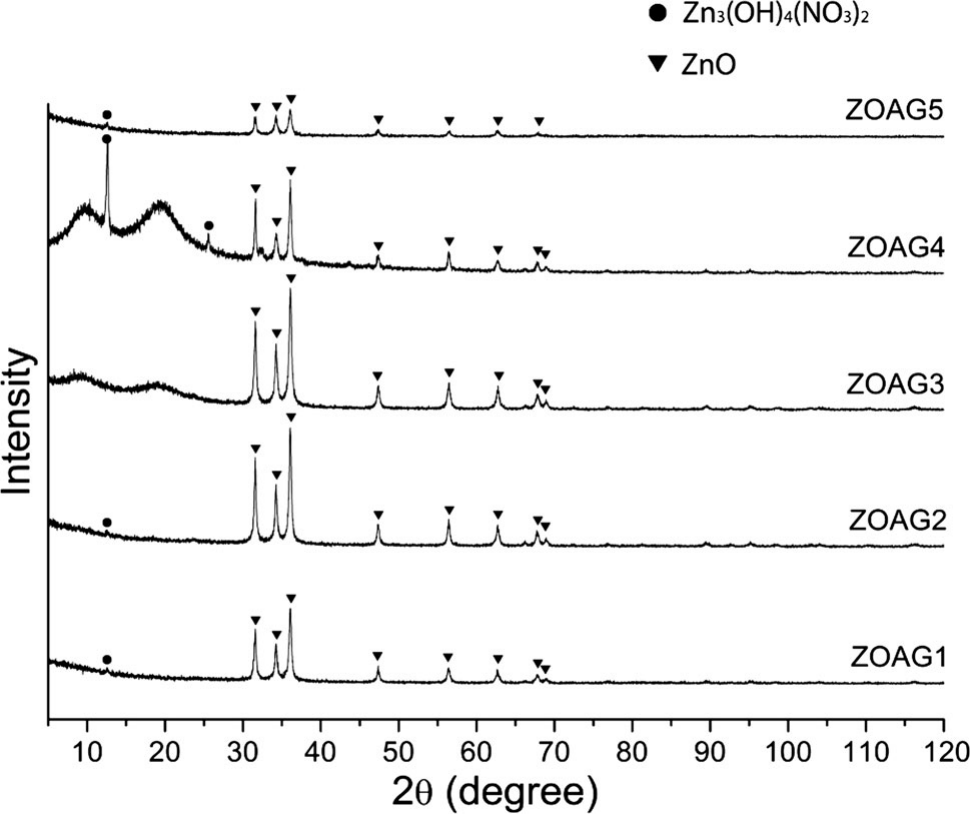
X-ray diffraction of ambient pressure dried porous ZnO composites ZOAG1–ZOAG5

The XRD results of the samples after being heat-treated at 200 °C are presented in Fig. 5. The results show that all the samples ZOAG1–5 contain ZnO (PDF 01-079-0206). ZOAG4 contains also a new phase zinc hydroxide nitrate Zn3(OH)4(NO3)2 (PDF 01-070-1361) (Zn3) which is another type I structure of zinc hydroxide nitrates. In the XRD patterns of ZOAG4, the most prominent planes are at 12.5° and 25.5° of 2*θ* and are assigned to (100) and (200) planes of Zn3, respectively (PDF 01-070-1361). The samples ZOAG1, ZOAG2 and ZOAG5 have mostly ZnO phase with only a minute amount of Zn3 phase since only the leading diffraction plane (100) has been observed. In Fig. 5, we see that ZOAG3 has a pure ZnO phase. Therefore, the Zn3-based component is an intermediate product which occurs during formation of porous ZnO composites from porous Zn5-based composites and porous Zn1-based composites. Some materials can be directly formed from the sol–gel process without generating any intermediate product during drying such as silica and alumina [18, 22]. However, in this work various porous Zn-based (zinc hydroxide nitrate) composites are obtained by variation of drying conditions. In order to obtain porous ZnO composites, one has to do dehydration of porous Zn-based composites, the samples ZOAG1–3 are obtained from ZBAG1–3 after heat treatment at 200 °C, and they are imaged by TEM (Fig. 4d–f). The nanoplates are observed, but they are much smaller than that of ZBAG samples. The porous structures are also clearly observed in the TEM images of samples ZOAG 1–3 (Fig. 4d–f). The pore sizes distributions analysed by ImageJ of TEM images show the presence of micropores and mesopores (Fig. 4g–i). Comparing TEM images of samples ZOAG 1–3 (Fig. 4d–f) with samples ZBAG 1–3 (Fig. 4a–c), samples ZOAG show that the structure is less ordered, e.g. with shorter range order, the number and size of large rectangular nano-platelets is reduced. Additionally, ZOAG samples are more porous when compared to ZBAG samples. The dehydration of Zn-based nano-platelets induces an extensive microstructure transition with clear nanoporous platelets (Fig. 4g–i) that are assembled within ZnO particles. All pore sizes within the nano-platelets in ZOAG samples are similar, in range 1–3 nm, with maxima at ~ 2 nm in size.

ZnO particles size (*L*) can be estimated by Scherrer’s equation: *L* = *K λ*/(*B* cos*θ*), where *K* is shape factor, *B* is full width at half maximum (FWHM), *λ* is X-ray wavelength (0.1542 nm) and *θ* is diffraction angle. According to Bragg’s law, the *d* spacing can be calculated by expression, *λ*/2 sin*θ*. All our obtained ZnO samples have a wurtzite (hexagonal) crystal structure (*PDF 01*-*079*-*0206*). Therefore, the lattice parameters have a relation with d spacing as (1/*d*)^2^ = 4(*h*^2^ + *k*^2^ + *hk*)/3*a*^2^ + l^2^/*c*^2^. The XRD patterns of sample ZOAG1 have peaks with 2*θ* values of 47.36°, 56.40° and 62.66° corresponding to (102), (110) and (103) planes and FWHM values of 0.34, 0.40 and 0.43, by Scherrer’s equation, giving the particles sizes of 25.53, 22.55 and 21.65 nm, respectively. Therefore, an average size of ZnO can be estimated to be 23.2 nm. According to Bragg’s law, the d spacing is *d* (102) = 0.192 nm and *d* (110) = 0.163 nm. From planes (102) and (110), the lattice parameters of sample ZOAG1 were calculated by the relation of the lattice parameters as *a* = *b* = 0.326 nm and *c* = 0.524 nm. Similarly as above, samples ZOAG2 and ZOAG3 have average sizes, 26.0 and 21.3 nm, respectively. The lattice parameters of ZOAG2 and ZOAG3 are all *a* = *b* = 0.326 nm and *c* = 0.524 nm. Samples ZOAG 1–3 have a same ratio of lattice parameters *c*/*a* to be 1.607 which is close to the ideal hexagonal unit cell (*c*/*a* = 1.633), and the Zn–O length calculated from geometry of the ZnO wurtzite crystal structure is 0.1986 nm. Interestingly, porous ZnO samples with wurzite (hexagonal) crystal structure are promising materials in energy applications, due to their pyroelectric properties [4].

**Figure 6:**
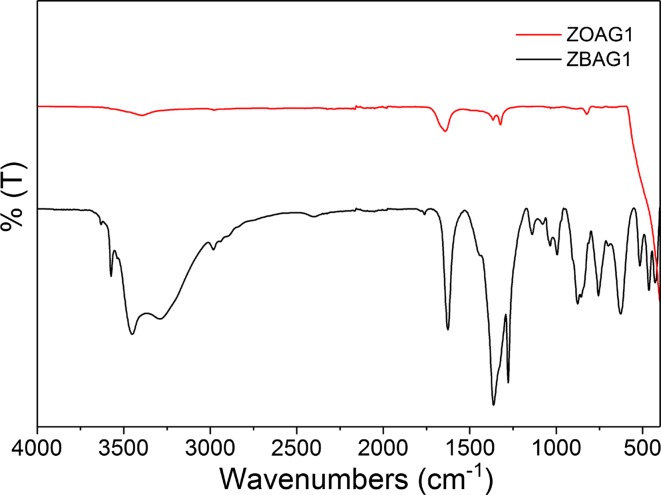
FTIR of samples ZBAG1 and AOZG1

In order to study the chemical composition of the products, samples ZBAG1 and ZOAG1 were characterised by FTIR. The FTIR spectrum of sample ZBAG1 (Fig. 6) shows a broad band around 3300 cm^−1^ and a peak around 1630 cm^−1^ that corresponds to O–H stretching and H–O–H bending of Zn_5_(OH)_8_(NO_3_)_2_·2H_2_O, respectively [34], and further band at ~ 1363 cm^−1^ corresponds to NO_3_ vibrations in Zn–NO_3_ [35]. The FTIR spectrum of sample ZOAG1 shows the most prominent band is observed in energy range 400–500 cm^−1^, which corresponds to Zn–O stretch [36]. In addition, comparing the FTIR spectrum of ZOAG1 with that of ZBAG1, there is absence/weakness of peaks observed in ZBAG1 sample over all energy range; therefore, this confirms the phase transition from Zn_5_(OH)_8_(NO_3_)_2_·2H_2_O to ZnO upon heat treatment. The successful fabrication and purity of ZnO via our APD method is comparable to SCD method published in the literature [4, 5] showing advantages in manufacturing process with reduced equipment requirements.

**Figure 7:**
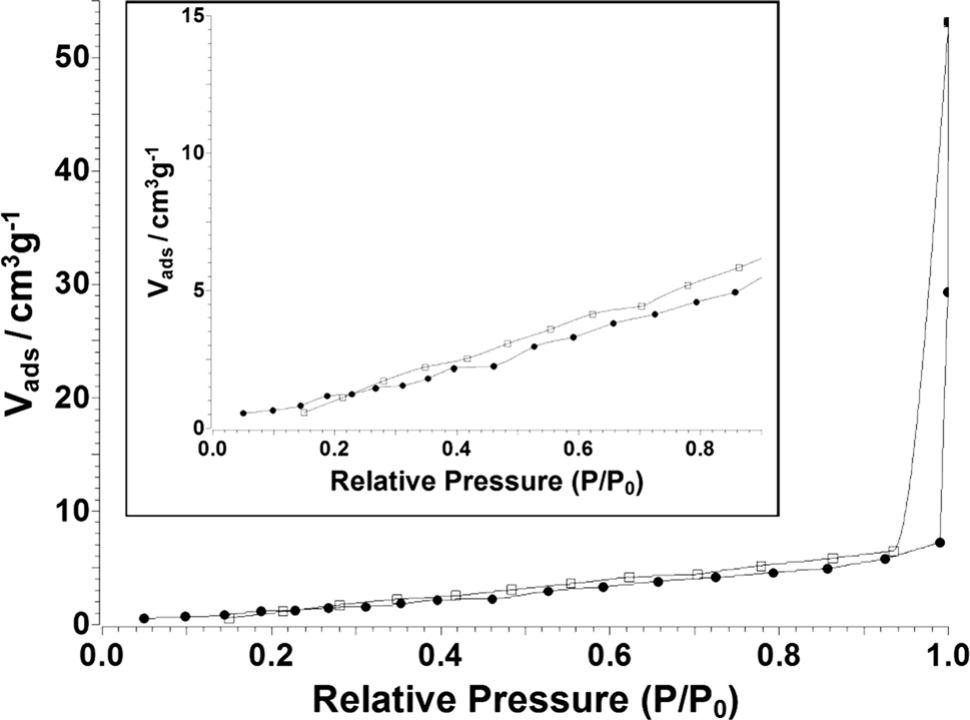
Nitrogen adsorption–desorption isotherm of sample ZBAG 1

The ZBAG 1 sample, which has the single phase of porous Zn_5_-based product, was also characterised by nitrogen gas adsorption and desorption isotherm at 77 K (Fig. 7). According to the classification of isotherm types determined by the International Union of Pure and Applied Chemistry (IUPAC), a standard type VI isotherm is identified, and it represents a typical macroporous material with highly uniform surfaces [37]. This is in agreement with the flower-like macroporous architecture revealed in Fig. 3a–e which consists of large rectangular nanosheets (Fig. 4a–c). The surface area of ZBAG 1 is 7.26 m^2^/g which also verifies the macroporous structure of the sample. The surface area of ZOAG 1 is 11.80 m^2^/g, showing increase in surface area upon heating. We suggest that the increase in surface area from porous Zn-based composites to porous ZnO composites is caused by the change of microstructure as observed by the transmission electron microscope images, as comparison between Fig. 4a–f. It is known [38] that if the pore size is very small or comparable with diameter of adsorptive molecules used in isotherm adsorption measurements, surface areas will be unreliable and can be underestimated. Possible reasons are [38]: (1) that there is a different density of adsorbed molecules during pore filling, in small pores comparing to usual liquid phase, which is known to be used as standard input parameter in adsorption isotherm analysis method (such as BET); (2) the roughness of the materials cage containing the pores with pore shapes influencing the adsorption: (3) changes in effective cross-sectional area of adsorptive molecules. In case of N_2_, N_2_ has also a quadrupole moment, so the electrostatic repulsion of N_2_ with ZnO/Zn-based cages during adsorption process within the small pores (< 2 nm) may give underestimated surface area of the material.

Finally, the majority of porous planes are self-assembled in stacks to form flower-like microstructure (Fig. 3f), so the diffusion of nitrogen into the stack of porous planes during N_2_ adsorption experiment may be hindered, which would give an underestimate of the sample surface area. Further study of adsorption of different adsorptive molecules (such as argon, CO_2_) at different temperatures and with different methods is required, to understand pore adsorption and pore size distribution and distinguish above-mentioned phenomena.

**Figure 8:**
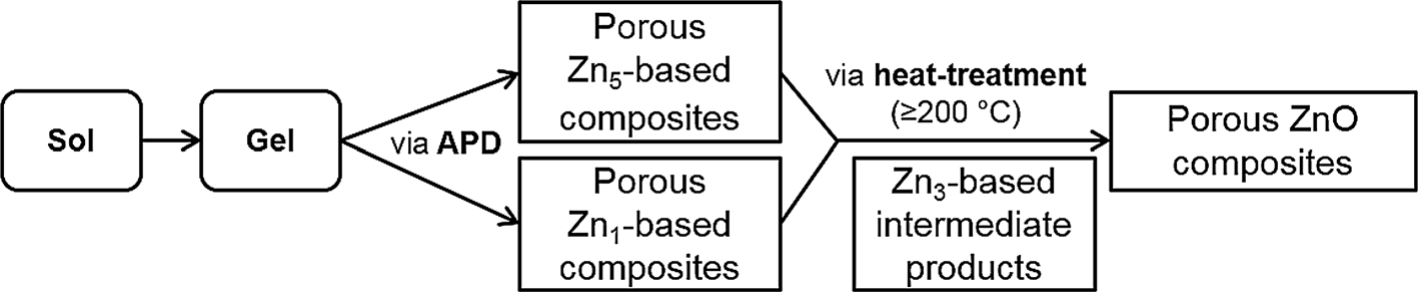
Schematic diagram of ambient pressure drying routes of porous Zn-based/ZnO composites

In APD synthesis in this work, porous ZnO composites can be only obtained after additional heat treatment after the initial synthesis of porous Zn-based composites is completed. Therefore, it is essential to know the components of the materials at each stage for the APD synthesis. We suggest a process presented in Fig. 8 for control and design of the APD synthesis of porous Zn-based/ZnO composites.

## Conclusions

Porous Zn-based and ZnO composites are successfully fabricated via the sol–gel process and ambient pressure drying route. The variations of phases are identified by the XRD analysis. When porous Zn-based composites are obtained, high drying temperature (200 °C) and large casting mould size are required to obtain Zn_1_-based samples. Alternatively, low drying temperature and small casting mould size are required for Zn_5_-based samples. Morphologies of different phases are studied by SEM and TEM. A macroporous flower-like structure containing the nanosheets is observed in Zn-based products by SEM imaging. Micropores and mesopores in ZnO aerogels are observed in the TEM images. This work reports conditions necessary for the ambient pressure dried ZnO porous materials and may be important for future synthesis of ZnO porous material composites (with silica, alumina, etc.) using the APD method. The APD synthesis proposed has low energy requirements (only heating at 200 °C at atmospheric pressure) and does not require expensive equipment as in SCD.

## Electronic supplementary material

Below is the link to the electronic supplementary material.

Supplementary material 1 (DOCX 313 kb)
