# Supplementary material for: Synthesis of porous zinc-based/zinc oxide composites via sol–gel and ambient pressure drying routes
Source: J Mater Sci. 2018 Feb 26;53(11):8170–9. doi: 10.1007/s10853-018-2138-2 (PMC6566210; doi:10.1007/s10853-018-2138-2)
Supplement: Supplementary file 1 — Supplementary material 1 (DOCX 313 kb) [file 10853_2018_2138_MOESM1_ESM.docx]

Supporting Information

Synthesis of Porous Zinc-Based/Zinc Oxide Composites via Sol-Gel and Ambient Pressure Drying Routes

Xiao Han, Jonathan Harris and Lidija Šiller *

School of Chemical Engineering and Advanced Materials, Newcastle University, Newcastle upon Tyne NE1 7RU, UK

[Xiao.Han@newcastle.ac.uk](mailto:Xiao.Han@newcastle.ac.uk); [J.Harris2@newcastle.ac.uk](mailto:J.Harris2@newcastle.ac.uk); [Lidija.Siller@newcastle.ac.uk](mailto:Lidija.Siller@newcastle.ac.uk)

The repeating experiments ZBAG R1-R3 and R’1-R’3 (Table S1) utilised the exact same process as those of ZBAG 1-3. The samples were characterised by XRD as shown in Fig. S1. which verifies the results of ZBAG 1-3 in the manuscript.

**Table S1**

Conditions of preparing ambient pressure dried Zn-based (ZBAG) and ZnO (ZOAG) porous composites in this work.

| Sample | Diameter of mold (mm) | Ageing solvent | Initial drying temperature (°C) | Heat treatment (°C) |
| --- | --- | --- | --- | --- |
| ZBAG R1 and R’1 | 11 | Methanol | 60 | - |
| ZBAG R2 and R’2 | 11 | Methanol | 65 | - |
| ZBAG R3 and R’3 | 9 | Methanol | 60 | - |


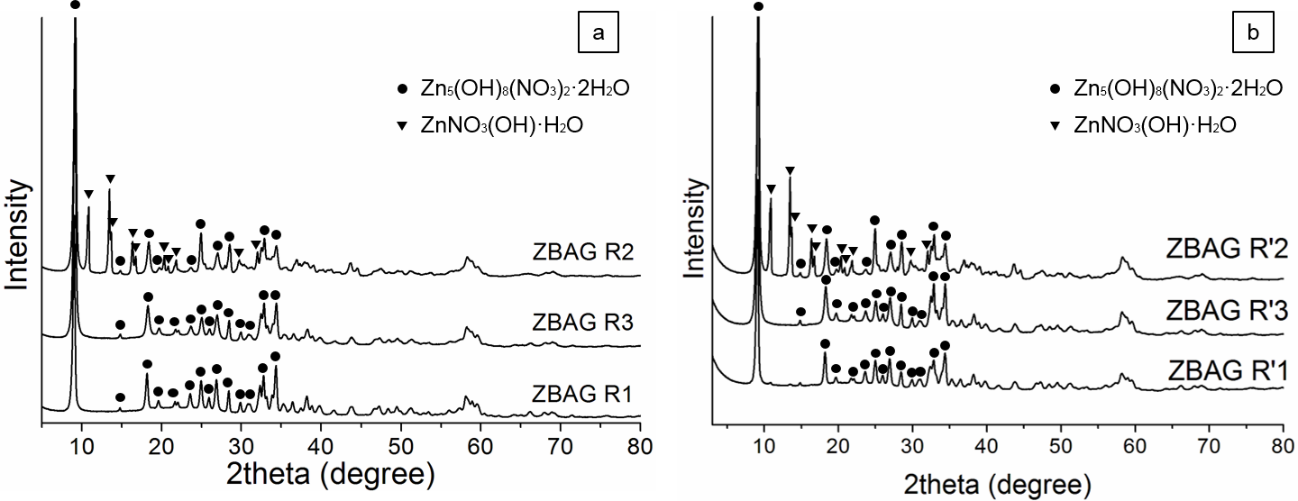


**Fig. S1.** XRD of samples a) ZBAG R1-R3 and b) R'1-R'3.
